# Motor Control System for Adaptation of Healthy Individuals and Recovery of Poststroke Patients: A Case Study on Muscle Synergies

**DOI:** 10.1155/2019/8586416

**Published:** 2019-03-27

**Authors:** Fady S. Alnajjar, Juan C. Moreno, Ken-ichi Ozaki, Izumi Kondo, Shingo Shimoda

**Affiliations:** ^1^College of Information Technology (CIT), The United Arab Emirates University, Al Ain, UAE; ^2^RIKEN BSI-Toyota Collaboration Center, Aichi, Japan; ^3^Neural Rehabilitation Group, Cajal Institute, National Council for Scientific Research (CSIC), Madrid, Spain; ^4^National Center for Geriatrics and Gerontology, Aichi, Japan

## Abstract

Understanding the complex neuromuscular strategies underlying behavioral adaptation in healthy individuals and motor recovery after brain damage is essential for gaining fundamental knowledge on the motor control system. Relying on the concept of muscle synergy, which indicates the number of coordinated muscles needed to accomplish specific movements, we investigated behavioral adaptation in nine healthy participants who were introduced to a familiar environment and unfamiliar environment. We then compared the resulting computed muscle synergies with those observed in 10 moderate-stroke survivors throughout an 11-week motor recovery period. Our results revealed that computed muscle synergy characteristics changed after healthy participants were introduced to the unfamiliar environment, compared with those initially observed in the familiar environment, and exhibited an increased neural response to unpredictable inputs. The altered neural activities dramatically adjusted through behavior training to suit the unfamiliar environment requirements. Interestingly, we observed similar neuromuscular behaviors in patients with moderate stroke during the follow-up period of their motor recovery. This similarity suggests that the underlying neuromuscular strategies for adapting to an unfamiliar environment are comparable to those used for the recovery of motor function after stroke. Both mechanisms can be considered as a recall of neural pathways derived from preexisting muscle synergies, already encoded by the brain's internal model. Our results provide further insight on the fundamental principles of motor control and thus can guide the future development of poststroke therapies.

## 1. Introduction

Behavioral adaptation to unpredictable environmental changes is one of the most powerful capabilities for performing activities of daily living (ADL). For example, we can walk not only on flat asphalt but also on gravel and we can easily move from the living room to the kitchen no matter how the furniture is arranged or even how frequently it is rearranged. Moreover, behavioral adaptation has been found to be essential for enhancing the quality of communication between people [1]. Thus, without adaptability to our surroundings, we would be unable to complete even simple ADL. Clarifying the computational mechanisms that underlie behavioral adaptation is necessary for understanding the fundamental principles of the motor control system and developing treatment for related disorders.

There have been many attempts to explain behavioral adaptation in humans, by analyzing neuronal activity [2–7] and biological control architectures [8–11] and by proposing learning mechanisms based on biological systems [12–16]. However, the computational mechanism that governs behavioral adaptation remains unsolved.

From the viewpoint of behavioral adaptation, the redundancy of the musculoskeletal system plays a leading role in adjusting our behavior to the environment, as Bernstein pointed out half a century ago [17]. For instance, even the relatively simple motion of the shoulder joint is achieved by the complex combination of at least nine muscles [18]. Investigating how the central nervous system (CNS) groups and recruits muscles depending on the task and knowledge of the surrounding environment may provide a fundamental clue behind neuromuscular adaptability.

To explain the plausible biological computational mechanism for choosing the appropriate combination of muscles to control point-to-point movements, several researchers have proposed the notion of muscle synergy [19–23]. Muscle synergy, defined as the relative weight of muscle activations driven by common excitation primitives, provides a simple control algorithm, yet allowing for complex motor behavior [7].

Some motor characteristics of human behavior could be deciphered when behaviors were analyzed based on muscle synergy [24, 25]. In our previous study [26], we introduced two indices, based on muscle synergies, and experimentally showed that they represent the efficiency by which basic movement skills (e.g., upright balance skills) are adapted to the environment. The purpose of the current study was to determine whether neuromuscular control strategies are comparable between healthy individuals during their adaptation to an unfamiliar environment and stroke survivors during their recovery. Note that we do not compare here how the muscle synergy pattern is shaped in each case but how the increasing or decreasing of muscle synergy dimensionality is similar between adaptation/recovery. This similarity could be related to the model adopted by the CNS to represent how much it knows about the environment. We considered that this would provide crucial insight and better understanding for developing poststroke rehabilitation systems, better tailored to the treatment of specific motor deficits. To facilitate this study, we have focused on investigating the muscle synergy adaptability of a well-understood single-joint motor task, such as shoulder flexion, which was introduced to the poststroke patients and shoulder adduction introduced to the healthy participants [27]. The reason for presenting two different types of movements to each type of participants is due to the nature level of the participants. Shoulder flexion task was introduced to the patients due to their constrained range of joint motion. For healthy participants, on the other hand, shoulder adduction was introduced due to limitation of the need of using robotic manipulandum capable to produce hard enough task to stimulate their muscle synergy adaptation. Despite focusing on this task, we believe in the generalization of synergy features [28].

We hypothesized that, in poststroke patients with motor dysfunctions, the brain responds as if experiencing an unknown environment due to the interruption of established neural pathways, similar to what occurs when healthy individuals experience an unfamiliar environment. We also argue that the use of muscle synergy analysis for clarifying the pathology of poststroke patients with unilateral motor impairment could provide greater diagnostic accuracy and may help to design more effective poststroke rehabilitation programs than using common clinical tests, which cannot illustrate progress at a neural level during rehabilitation [29–32].

In this study, we compared the behaviors of poststroke patients during an eleven-week recovery phase with those of healthy participants during performance of movements in familiar and unfamiliar environments/tasks. We also analyzed changes in muscle synergy in both scenarios.

## 2. Materials and Methods

### 2.1. Experimental Setup and Protocol

#### 2.1.1. Healthy Participants: Experimental Setup

*(1) Participants.* Nine healthy adults (age: 38.1 ± 7.8 years (mean ± standard deviation (SD))) participated in this experiment. All participants were right handed and reported no neurological or upper limb muscular impairments. The experimental protocol was approved by the RIKEN ethics committee. Written informed consent was obtained from all participants.

**Figure 1:**
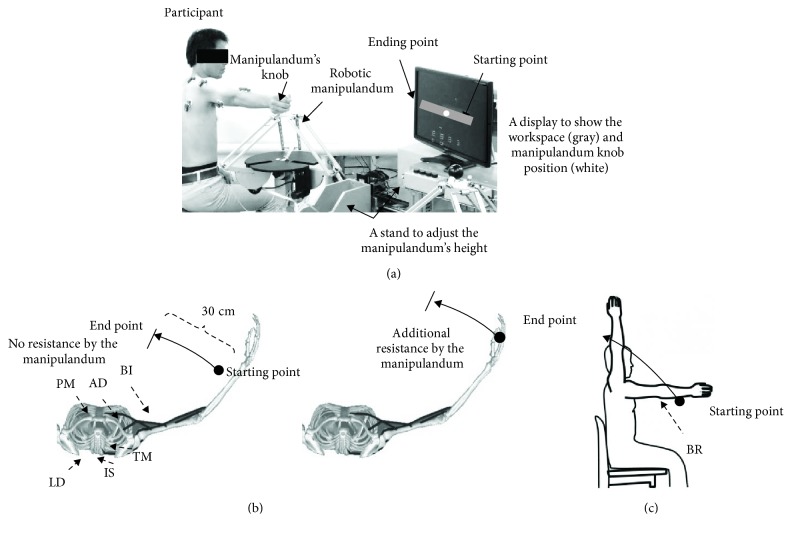
Experimental setup and protocol for healthy participants (more details are in [37]). (a) Participant posture, manipulandum, display positions, and workspace (30 cm long). The white circle in the display illustrates the position of the manipulandum knob in the space. The knob position was displayed to the participant to simplify tracking in the assigned task (the display moved horizontally with the knob from the starting point to the ending point of the workspace). (b) Top view illustrating the relevant muscles in the upper torso and right arm in the two tasks for the healthy group—left: the task in the standard environment; right: the task in the modified and adaptation environment. *Standard environment:* move the knob from the starting point to the ending point 10 times (no resistance applied). *Modified environment:* move the knob from the starting point to the ending point 20 times (no resistance and various resistances applied randomly). *Adaptation environment:* move the knob from the starting point to the ending point 15 times for two sessions separated by resting time (7-N resistance applied by the manipulandum at all trials). PM: pectoralis major; AD: deltoid anterior: IS, infraspinatus; TM: teres major; LD: latissimus dorsi; BI: biceps brachii. (c) Side view illustrating the task for the poststroke group. Five muscles were recorded in both the intact and the affected shoulder. Muscles that were shared with healthy participants are the PM, AD, IS, and BI. While BR, brachioradialis, was newly introduced to accommodate the task.

*(2) Robotic Manipulandum*. Participants were asked to control the gripper position of a robotic manipulandum (Force Dimension, Nyon, Switzerland) by using their right arm (see Figure 1(a)). The manipulandum contains a bilateral control system, allowing participants to experience the computer-generated virtual space through the forces generated. The robot manipulandum was used to constrain motion and generate an “unfamiliar” environment to healthy participants, by introducing random stiffness “resistance” to the arm movement, in various levels; three types of resistance were used: 4, 7, and 10 N. During the experiment, participants were seated on an adjustable chair with the right hand holding the knob of the manipulandum from the side. A monitor display showing the virtual space was placed in front of the participant to provide feedback for the performance on the assigned tasks.

*(3) Electromyography (EMG)*. Six surface EMG electrodes were placed on the participant's right shoulder to record the activity of primary muscles. Due to the nature of the assigned task, comprising right hand horizontal shoulder adduction, the following muscles were recorded: pectoralis major (PM), deltoid anterior (AD), infraspinatus (IS), teres major (TM), latissimus dorsi (LD), and biceps brachii (BI). EMG electrodes were positioned in accordance with the guidelines of Surface EMG for the Non-Invasive Assessment of Muscles—European Community project [33]. EMG signals were sampled at 1 kHz, high-pass filtered with a cutoff frequency of 30 Hz, root-mean-square rectified, and smoothed using a moving average with a window length of 10 samples. The EMG from each muscle was normalized to its peak value from the experimental set. EMG data were synchronized with the manipulandum data through the use of a common clock and trigger.

#### 2.1.2. Healthy Participants: Experiment Protocol

The participants were asked to perform tasks in three different environments:

1. *Standard Environment*. We asked each participant to grasp the knob of the manipulandum and perform 10 trials of horizontal shoulder adduction from a predefined starting position (see Figure 1(b)). To ensure that the experimental constraints were similar across individuals, we instructed participants not to use their elbow, to maintain a constant contraction of their shoulder muscles, and to complete each trial in 1 s. In this environment, the manipulandum did not produce any significant resistance on the participant's arm. The starting and ending points of the movement were denoted by the manipulandum knob position and displayed on the screen in front of the participants. Particularly for this environment, participants were asked to perform 20 practice trials for familiarizing themselves with the environment before the start of data collection
2. *Modified Environment*. To introduce an unfamiliar environment to the participants, we modified the manipulandum responses, compared to those used in the standard environment. In this environment, the manipulandum randomly applied resistance varying from 4 ~ 10 N on the participant's arm, opposite to the movement's direction (see Figure 1(b)). The resistances were applied randomly. Thus, participants could not predict the resistance, thereby reducing the possibility of adaptation at this stage. We asked each participant to repeat the horizontal shoulder adduction task 20 times
3. *Adaptation Environment*. Here, we examined the participants' adaptability to the unfamiliar environment through training. We determined this environment as unfamiliar, because even healthy participants could not master the movement in a single trial [34]. At this stage, we asked participants to perform 30 trials of horizontal shoulder adduction. The participants were given a rest period of 120 s after the first 15 trials to minimize muscle fatigue. In this environment, a resistance of 7 N was applied continuously for all trials (although we have done a pilot of resistance varying from 4, 7, to 10 N, the 7 N resistance is the one which we could see little change on the initial computed muscle synergy. The 4 N resistance showed no muscle synergy changes, and in the 10 N resistance, it was hard for the participant to adapt due to muscle fatigue)

#### 2.1.3. Poststroke Patients: Experimental Setup

*(1) Participants*. Ten poststroke patients (age: 66.5 ± 11.6 years (mean ± SD)) participated in this experiment (see Table 1). All patients were recruited 1.5~2 months after stroke onset and were diagnosed with moderate unilateral motor impairment according to the stroke impairment assessment set (SIAS) (score, 2-4 out of 5) [35, 36]. The experiment protocol for stroke patients was approved by the ethics committee of the National Center for Geriatrics and Gerontology, Aichi, Japan.

*(2) Electromyography*. Surface EMG was recorded from muscles of the patient's affected and intact shoulders, while performing a shoulder flexion task, as described below. Five primary muscles were recorded in each shoulder: PM, AD, IS, BI, and brachioradialis (BR).

*(3) Experiment Protocol*. Due to the nature of the patients' impairment and their constrained range of joint motion, we asked the patients to do a simple bimanual shoulder flexion task. This was to compare muscle synergy in the intact and affected arms of the same patient, instead of just comparing it with that in the arm of a healthy individual. A set of 10 to 15 trials for each session was conducted by each patient, to avoid fatigue (see Figure 1(c)).

### 2.2. Muscle Synergy Computation

Muscle synergy has been described as a systematization method by which some muscles are activated in synchrony to complete a task [22, 37]. It defines how muscles are synchronized using the following fixed matrix:

(1) $$\begin{array}{c} M=WC,\\ M\in R^{m\times t},\\ W\in R^{m\times n},\\ C\in R^{n\times t}. \end{array}$$

Here, *M* refers to the time sequence signals activating *m* muscles and *t* is the length of the time sequence. *W* is the fixed matrix defining the synchronization of *m* muscles. *n* represents the number of synergies and should be smaller than *m*. *W* is normalized as

(2) $$\begin{array}{c} W=\left[W^{\left(1\right)}W^{\left(2\right)}W^{\left(3\right)}\cdots W^{\left(n\right)}\right],\\ \left|W^{\left(i\right)}\right|=1, \end{array}$$

where *W*^(*i*)^ denotes the vector of size, expressed as

(3) $$\begin{array}{c} W^{\left(i\right)}\in R^{m}. \end{array}$$

**Figure 2:**
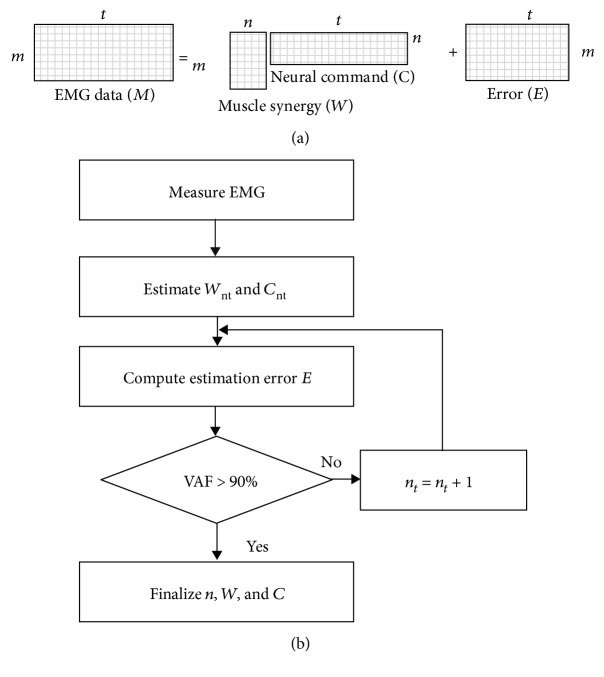
(a) A conceptual-mathematical model for identifying muscle synergies. (b) A flowchart that illustrates the process to estimate *n*, *W*, and *C*.

We refer to *W* as the *synergy space*. *C* refers to the control signal activating *m* muscles. Note that *n* time sequence signals in *C* are changed to *m* signals in *M* using matrix *W* in equation (1). Therefore, the dimensionality for controlling *m* muscles is reduced from *m* to *n* using this system. The conceptual image of this signal transformation is shown in Figure 2(a).

We can estimate *W* and *C* from recorded EMG data using nonnegative matrix factorization (NMF) [38]. The synergy dimension (SyD) of the neural signal *n* is one of the important parameters in determining the characteristics of muscle synergy. An appropriate *n* must be chosen according to the behavior, to estimate *W* and *C*. Thus, we chose *n* using the following steps (see also the flowchart in Figure 2(b)):

- Acquisition of EMG data for *m* muscles and generation of time sequence data for muscle activations (*M*) by filtering the raw EMG data
- Temporary definition of *n* as *n*_*t*_ and estimation of *W*_*nt*_ and *C*_*nt*_ using NMF
- Computation of the estimation error *E* as

(4) $$\begin{array}{c} E=M-W_{nt}C_{nt} \end{array}$$

(iv) Computation of the size of *E*, i.e., the variance accounted for (VAF), such that

(5) $$\begin{array}{c} \text{VAF}=1-\frac{{\left\|E\right\|}_{\text{F}}^{2}}{{\left\|M\right\|}_{\text{F}}^{2}}, \end{array}$$

where ‖.‖_F_ denotes the Frobenius norm. If VAF was smaller than a predefined threshold, we changed *n*_*t*_ to (*n*_*t*_ + 1) and computed *W*_*nt*_ and *C*_*nt*_ again by NMF. VAF increases as *n*_*t*_ increases. The threshold is decided based on the behavior, but, in general, we used approximately 90% as a threshold, to indicate a good fit to the original data [38]. By such a threshold, we guarantee that each recorded muscle curve would be well reconstructed

The computation is continued by increasing *n*_*t*_ to (*n*_*t*_ + 1) until VAF becomes larger than the threshold. We used the value of *n*_*t*_ + I as the dimension of the neural signal, thereby completing our selection of *n* and estimation of *W* and *C*.

### 2.3. Behavior Analysis Using Muscle Synergy

Both *W* and *C* represent interesting features of human motor behavior, as reported by Safavynia et al. [38]. Bizzi et al. [39] showed that *W* is not specific to individuals but is specific to behavior. Cheung et al. [40] analyzed muscle synergies in stroke survivors and showed that, at the beginning of recovery, some dimensions in synergy space *W* of the affected arm were merged, in comparison with those of the intact arm.

To analyze behavior by muscle synergy, we asked participants to repeat the assigned task several times (20-30 in healthy participants, 10-15 in stroke patients) and then computed *n*, *W*, and *C* for each trial, considering the time between the starting/ending of the movement (approximately 1.5 s). By comparing these parameters between trials, we derived the features of the behaviors. In our previous study [26], we introduced indices of similar *W* and *C* at each trial and showed that they represent the ability of automatic posture response in healthy participants. Here, we used the same method for computing *n*, *W*, and *C*, to identify changes in behavior during adaptation to an unknown environment. In the case of adaptation analysis, *n* represents the level of adaptation to the environment.

## 3. Results

### 3.1. Healthy Participants

All healthy participants completed the assigned tasks successfully.

#### 3.1.1. Dimensions of Synergy Space

**Figure 3:**
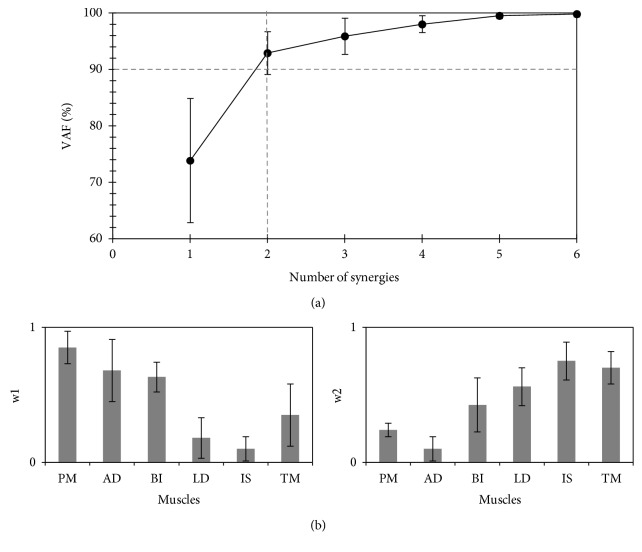
Synergy space at the standard (familiar) environment. (a) The variance accounted for (VAF) (%) all possible identified synergies from the recorded electromyograph while performing the task in the standard environment (mean ± SD, 9 participants). The dashed vertical line identifies the estimated number of utilized synergies that exceeded the threshold (90%; represented by the horizontal dashed line). (b) Muscle synergy vectors (*W*) for two-dimensional muscle synergies (*SyD.2*; mean ± SD, 9 participants). The orders of w1 and w2 were sorted based on their activation time (*C*). PM: pectoralis major; AD: deltoid anterior; BI: biceps brachii; LD: latissimus dorsi; IS: infraspinatus; TM: teres major.

*(1) Standard Environment*.  Figure 3(a) shows the dimensionality of the resulting muscle synergies in participants performing the task in the standard environment. All participants needed two-dimensional muscle synergies to complete the task (i.e., the VAF SyD.2 was the minimum number of synergies that exceeded the assigned >90% threshold). The functional role of each synergy is illustrated in Figure 3(b): Synergy #1 (w1) seems to mainly be involved in activating the prime mover muscles (PM and AD), which are primarily responsible for shoulder adduction. In contrast, Synergy #2 (w2) seems to involve the manipulation of neutralizer muscles (BI, LD, IS, and TM), which assist the internal rotation of the shoulder joint and are essential to complete the desired shoulder adduction.

**Figure 4:**
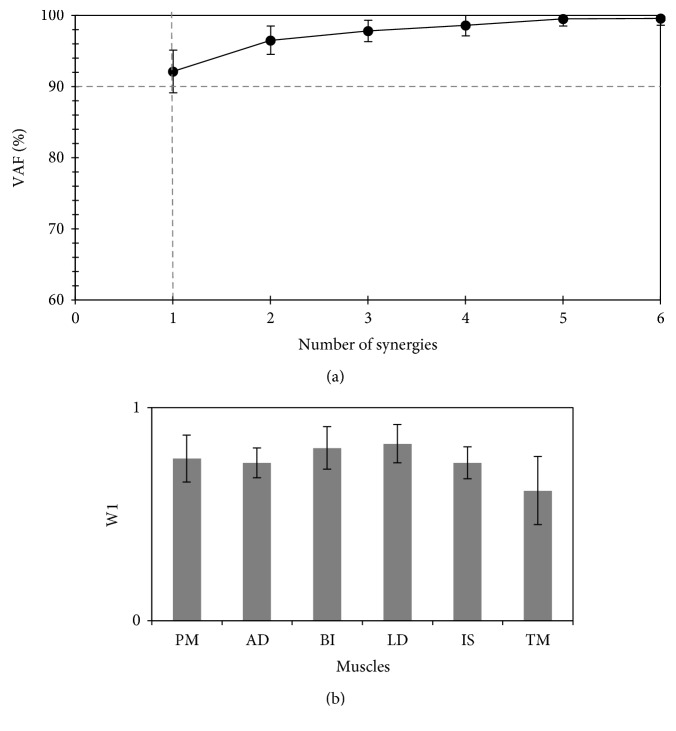
Synergy space at the disturbed (unfamiliar) environment. (a) The variance accounted for (VAF) (%) all possible identified synergies from the recorded electromyograph while performing the task in the *modified environment* (mean ± SD, 9 participants). The dashed vertical line identifies the estimated number of utilized synergies that exceeded the threshold (90%; represented by the horizontal dashed line). (b) Muscle synergy vectors (*W*) for one-dimensional synergy, *SyD.1*. PM: pectoralis major; AD: deltoid anterior; BI: biceps brachii; LD: latissimus dorsi; IS: infraspinatus; TM: teres major.

*(2) Modified Environment*.  Figure 4(a) shows the dimensionality of the resulting muscle synergies during task performance in the modified environment. When resistance was randomly applied, one-dimensional muscle synergy, on average, was observed in all participants for completing the task, instead of the original two-dimensional synergies. The identified synergy in this environment seemed to involve both the prime mover and neutralizer muscles (see Figure 4(b)), which could, in turn, have led to the reduction in the range of shoulder joint internal rotation, making the movement uncomfortable (participants reported of being tired after a few movements).

**Figure 5:**
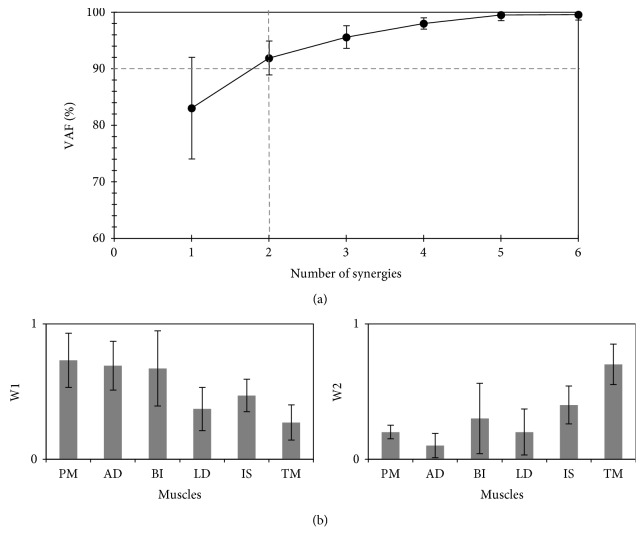
Synergy space after being adapted to the unfamiliar environment. (a) The variance accounted for (VAF) all possible identified synergies from the recorded electromyograph while performing the task in the *adaptation environment* (mean ± SD, 9 participants). The dashed vertical line identifies the estimated number of utilized synergies that exceeded the threshold (90%; represented by the horizontal dashed line). (b) Muscle synergy vectors (*W*) in the two- dimensional synergies, *SyD.2*. PM: pectoralis major; AD: deltoid anterior; BI: biceps brachii; LD: latissimus dorsi; IS: infraspinatus; TM: teres major.

*(3) Adaptation Environment*.  Figure 5(a) shows the dimensionality of the resulting muscle synergies in participants while performing the last 15 trials of the task in the modified environment (resistance was applied continuously). After behavioral adaptation to the unfamiliar environment, all participants returned to using two-dimensional muscle synergies to complete the task. The resulting synergies were similar in function to those observed in the standard environment, i.e., w1 and w2, which appeared to activate the prime mover and neutralizer muscles, respectively (see Figure 5(b)).

**Figure 6:**
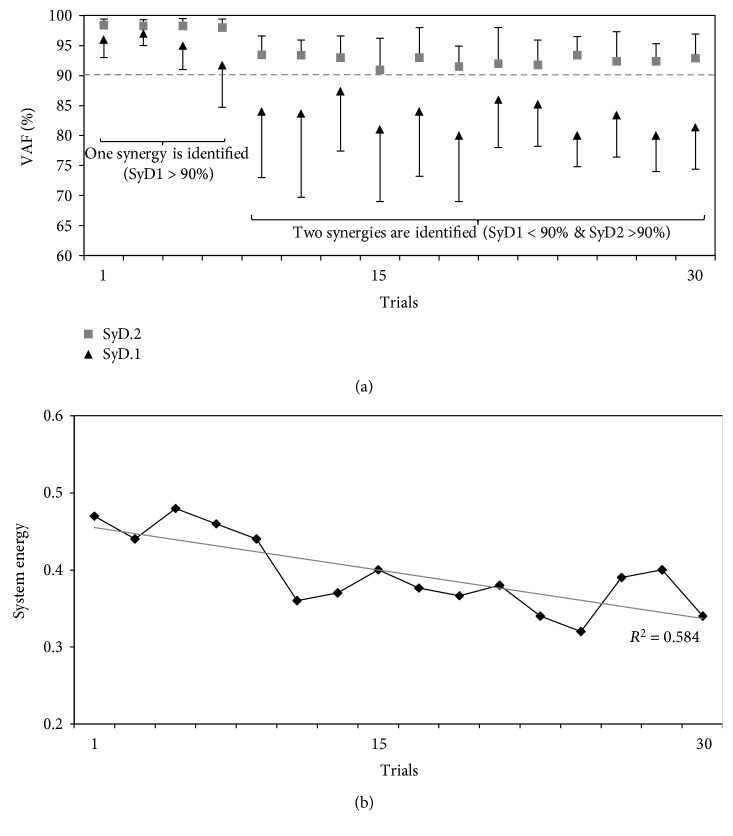
A gradual reduction in energy consumption as proper muscle synergies were recruited. (a) The gradual conversion of the one-dimensional synergy (SyD.1 > 90%) to two-dimensional synergies (SyD.1 < 90%) through training in the adaptive environment (mean ± SD, 9 participants). (b) Changes in system energy through adaptation in the *adaptation environment*. Linear least-squares regression line (*R*^2^ = 0.5841) to illustrate the adaptation direction. System energy was computed as the total muscle activation needed to complete a trial (mean, 9 participants). Greater muscle activations are associated with higher energy cost.

Figure 6(a) shows the gradual transformation from one-dimensional muscle synergy to two-dimensional muscle synergies over the 30 trials. The one-dimensional synergy (SyD.1) gradually decreased to below the threshold. After behavioral adaptation, the two-dimensional synergies (SyD.2) recovered to control the movement (SyD.2 > 90).

#### 3.1.2. Energy Consumption

To understand the mechanism of muscle synergy formation during adaptation by the CNS, we included the energy consumption calculations [41]. To investigate the changes in system energy consumption required to complete the task in the adaptation environment [42, 43], we measured the average total muscle activations over the trials (see Figure 6(b)). We found that, after approximately 10 trials, lower muscle activation was needed to complete the task, indicating that joint movements and muscle activations are gradually improved by task repetition through environmental interaction, thus minimizing energy consumption by the movement, by finding more efficient motor solutions, i.e., correct muscle synergy recruitments, to complete the task.

### 3.2. Poststroke Patients

All poststroke patients completed the assigned tasks successfully.

#### 3.2.1. Muscle Synergy Dimensionality for the Stroke-Affected and Intact Arms

**Figure 7:**
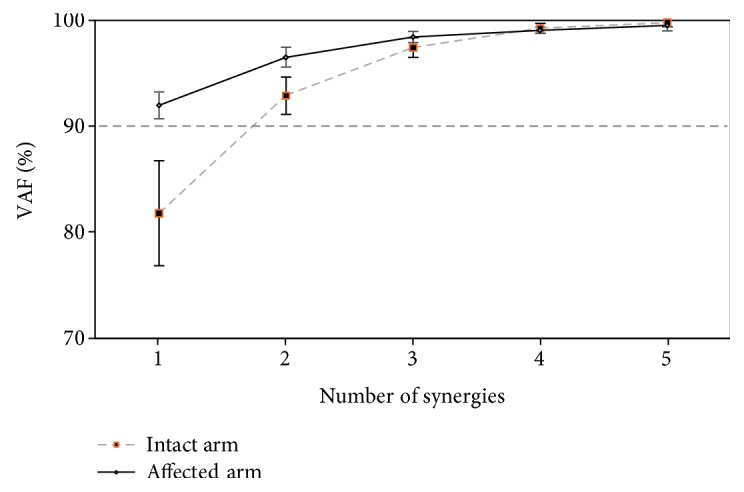
The variance accounted for (VAF) (%) all possible identified synergies from the recorded electromyograph while performing the task using the stroke-affected or intact arm, collected from ten patients with moderate stroke (mean ± SD). One-dimensional synergy (*SyD*.1 > 90%) is identified from the affected arm, while two-dimensional synergies (*SyD*.1 < 90% and *SyD*.2 > 90%) are identified from the intact arm of the patients.

Figure 7 shows the dimensionality of the resulting muscle synergies of the affected and intact arms of the 10 patients (mean ± SD). On average, one-dimensional synergy was used to produce motion in the affected arm, while two-dimensional synergies were used to produce motion in the intact arm.

Regarding the functionality of the resulting synergies on the intact arm, similar to the healthy participants, w1 was involved in activating the prime mover muscles, while w2 was involved in activating the neutralizer muscles. The one-dimensional synergy in the affected arm, however, seemed to activate all recorded muscles in synchrony, revealing an abnormal synergy [44].

**Figure 8:**
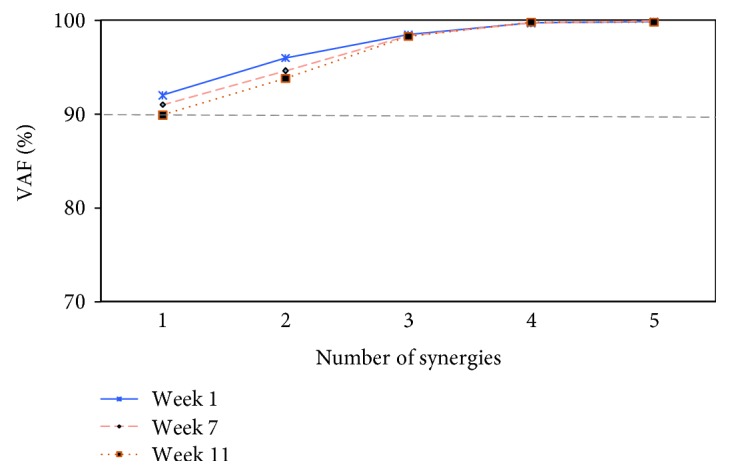
Muscle synergy dimensionality captures motor recovery. The variance accounted for (VAF) (%) all possible identified synergies from the recorded EMG while performing the task using the stroke-affected arm. Data were collected from 10 patients with moderate stroke (mean) at weeks: 1, 7, and 11 poststroke. The figure showing the gradual reduction of the one-dimensional synergy (SyD.1) towards the two-dimensional synergies (SyD.2) throughout the recorded period (mean ± SD), i.e., SyD = 1 has moved towards <90 VAF at week 11 (VAF = 89.8) compared to week 1 (VAF = 92). Thus, SyD = 2 became the representation of the recorded movement at week 11 instead of SyD = 1.

Figure 8 illustrates the adaptation process of the one-dimensional synergy (SyD.1) in all patients over the 11-week period in which they engaged in a regular rehabilitation program. Although the synergy remained one-dimensional, there were notable changes in the level of VAF, which resembled the formation of two synergies. Interestingly, these results indicate the gradual improvement in muscle recruitment in patients. Instead, evaluation by SIAS was unable to demonstrate this improvement along the test period for most patients (SIAS index still unchanged).

## 4. Discussion

The results of this study suggest that the CNS utilizes similar neuromuscular strategies both in the case of healthy individuals, when they adapt to an unfamiliar environment, and in that of poststroke patients, when they recover their motor function. Despite the energy inefficiency of movements produced by low-dimensional muscle synergies, the CNS seems to opt for this module at the initial stages of facing a new situation (or when the internal model is unable to predict appropriately the system output), to handle any unpredictable environmental inputs. By interacting with the environment, the CNS progressively learns to recruit more muscle synergies to conserve energy when it ascertains that the environment is now safe or, in other words, when it rebuilds enough internal model and is able to rely on it.

### 4.1. Features of Behavioral Adaptation in Healthy Participants

The experimental results in healthy participants revealed that the formation of muscle synergies is slightly altered when experiencing sudden changes in the familiar environment. The two-dimensional muscle synergies operating in the familiar environment were reduced to a single dimension when participants were first presented to the unfamiliar environment. However, this dimensionality reduction was accompanied by a simultaneous increase in muscle activations in response to the unfamiliar environmental inputs, suggesting that all muscles may be placed in a “standby” status, in order to promptly react to any unpredictable or unsafe input potentially occurring in the unseen environment/task, despite being energy inefficient. Nevertheless, our experiments show that training leads to a gradual adaptation to the new environment, resulting in the quick recovery of muscle synergy dimensionality to its original state. Moreover, the resulting energy consumption gradually decreases after the proper motor solutions are found. Note that the evaluation of the adaptation to the new environment, at this stage, was considered based on the computed muscle synergy of the healthy arm performing in a familiar environment.

The abovementioned findings are based on the assumption that movements required for the investigated task shared a commonality across humans. While it is true that for some specific tasks, muscle synergy vectors could vary between individuals, for example, a bench press task at different velocities, Samani and Kristiansen [45], the investigated task and perturbation experiments in this study have led us to conclude that muscle synergy vectors adequately account for the EMG activity associated with elbow movements in the pool of investigated subjects. This is also coherent with prior findings that reported stereotyped patterns of motor modules or synergies underlying the control of this motor task in healthy humans, demonstrating complete muscle patterns for specified arm movement task goals [23, 46].

### 4.2. Features of Motor Recovery after Stroke

Instead of directly examining muscle synergies, the VAF level can be used as it also seems to encode motor impairments. Our results in poststroke patients showed a gradual decline in the VAF over the recovery period. This could be interpreted as an effort by the CNS to optimize arm movement by tuning possible motor solutions, similar to what happens in healthy participants dealing with unfamiliar environments. Notably, some patients showed recovery in the number of recruited muscle synergies, i.e., from one- to two-dimensional muscle synergies, and their clinical score also improved. These results suggest that the dimensionality of muscle synergy can be used to measure the level of motor function recovery. A similar conclusion was deduced in a study by Cheung et al. [40]. Note that at this stage, the recovery level was evaluated based on the computed muscle synergy from the intact arm of the same patient performing the same motor task.

### 4.3. Neurophysiological Interpretations of Adaptation and Recovery

The question of whether muscle synergy during stroke recovery is newly constructed or simply adapted from existing synergies is a long-standing debate in neuroscience [47]. Our results here suggest that muscle synergies during recovery from moderate stroke most likely represent an adaptation of existing synergies, similar to what occurs in healthy individuals when neurons adapt to an unfamiliar environment.

In this study, we found that both motor adaptation in healthy participants and recovery in poststroke patients have comparable features regarding synergy dimensionality. Synergies in both cases varied as a function of the degree of control system adaptation to the environment. We postulate that, in healthy participants, the experience of the unfamiliar environment causes a temporal obstruction in CNS neural processes, as well as in muscles, which prevents the formation of efficient sets of muscle synergies, i.e., safety overcomes the efficiency. This can be inferred by the unregulated muscle activities and reduction in utilized muscle synergy dimensions. Similarly, in poststroke patients, lower dimension synergies operate in the stroke-affected arm than in the intact arm. Over time, however, we found that the dimensions of utilized synergies gradually increase in both healthy and poststroke participants, leading to the emergence of efficient motions.

### 4.4. Recruitment Strategies of Muscle Synergies and Internal Model Uncertainty

Behavioral studies have shown that the CNS employs various strategies during interaction with the environment to ensure the best possible protection for the body with the lowest possible energy consumption [48, 49]. Hypothetically, these strategies are mainly chosen depending on the accuracy of existing internal models representing the surrounding environment. For instance, accreditation to anticipatory movements is higher, when the internal model is properly trained and the environment is stable and predictable. In contrast, compensatory and energy consuming reflex movements increase, when the internal model is tacitly inaccurate due to an unstable environment [48]. Other behavioral studies on limb postural control have shown that the activities of muscles around a joint can be modulated to minimize the perturbing effects of unknown external loads [50, 51]. These modulations gradually decrease over the course of learning a novel motor task [48, 52].

The abovementioned literature findings are consistent with our results. In our experimental conditions, both the behaviors observed in the modified environment (healthy participants) and in the initial stage of rehabilitation (poststroke participants) can be regarded as pure compensatory movements in response to the new environmental condition, i.e., different dynamics of arm motion in healthy participants and different neural pathways in poststroke patients. However, these compensatory movements gradually change to anticipatory movements through training and interaction with the environment. The anticipatory movements correlate with the tuned muscle synergies, which interact efficiently with the familiar environment. In the unfamiliar environment, however, the simultaneous increase of muscle activities, although energetically expensive, may reflect a compensatory strategy to overcome the yet untrained internal model. These otherwise inefficient muscle activities gradually decrease over the course of interaction with the environment. In line with our findings, Kawato et al. [53] argued that the alteration of muscle activities when first learning new skills is effective in learning schemes that take advantage of motor command errors resulting from the feedback controller as learning signals during building of internal models.

### 4.5. Towards Neurorehabilitation

Currently, most muscle synergy studies are limited to offline synergy analysis, which focuses on classifying motor skill or impairment levels. To move beyond this stage and towards real application for rehabilitation, a better understanding of synergy usage during learning and adaptation is required. Testing various training hypotheses directly in poststroke patients can be a complicated task, due to the age of typical stroke patients and related factors. Our muscle synergy analysis results suggest that motor function recovery in poststroke patients is comparable to adaptation to unfamiliar environments in healthy participants. A natural next step would be to investigate the introduction of multiple unfamiliar tasks, i.e., build a stroke-like scenario in healthy participants, and test various training/rehabilitation protocols to determine ways to enhance the adaptation process, before using such protocols in poststroke patients.

In our protocol, we tried to avoid/reduce muscle fatigue; although this might not be fully possible, especially in the case of more demanding scenarios (e.g., during repetitive training tasks for poststroke treatment), it should be noted that muscle fatigue reduces strength and increases perceived effort, as observed in joint kinematics and movement complexity analyses in healthy individuals [54]. However, these changes due to muscle fatigue do not reflect alterations in the overall principal component shape [55, 56]. In contrast, our results are in agreement with prior results by Simkins et al. [57], demonstrating that differences between joint movements in pathological conditions are comparable to the differences observed for able-bodied movement synergies, further supporting the hypothesis that altered synergies upon neurological injury are an expression of similar spinal mechanisms, as those regulating intact synergies in multijoint movements. Furthermore, in their work, Simkins and colleagues [57] argue that alterations in pathological synergies during rehabilitation are shaped by plasticity at the spinal level. Interestingly, Jacobs et al. [58] discussed that, in tasks requiring high cortical involvement, the effect of training on the organization of intact muscle synergies is expressed with changes in modular organization, while in more basic, automated movements (e.g., walking), requiring less cortical activity, no changes in synergy number and structure are found. Equivalently, Torres-Oviedo et al. [22] investigated synergy organization during postural control (e.g., during walking) and showed that synergy robustness does not depend on reflex pathways or from biomechanical task constraints. In agreement with Krishnamoorthy et al. [59], our results demonstrate that muscle synergies and their organization are specific to the task, since they change with changes in stability conditions and new muscle synergies emerge to account for changes in postural responses.

## 5. Conclusion

The goal of our study was to explore the computational mechanism behind behavioral adaptation in humans when encountering an unfamiliar environment and how it compares to behavioral recovery in poststroke patients. Uncovering this mechanism would enhance our understanding of motor control and recovery and offer guidance to develop new rehabilitation approaches for various neural disorders. These results suggest that the CNS monitors the familiarity of the internal model with the surrounding environment and, relying on that, predicts the suitable motor control strategy by tuning muscle synergy dimensionality. When the internal models are immature, the CNS utilizes more muscles with high activities, by recruiting fewer synergies, to compensate for unexpected interactions with unfamiliar environments. These extra utilized muscles may work as an additional neural feedback to update the internal model. When learning occurs and the internal model representations are built up, the CNS decreases the movement energy by increasing the recruited muscle synergies.

We conclude that abnormal muscle patterns in poststroke patients are similar to the patterns observed at the beginning of neuronal network adaption in new environments. Changes in muscle synergy can be used as an indicator of motor function recovery, as indicated by our experiments in healthy participants and also supported by prior results as a valid source to design metrics to quantify acquisition of motor skills in healthy humans [26]. We are currently developing an advanced rehabilitation system with an online assistive robot that takes into account patient pathology and interindividual synergy variability to support motor function recovery. Future studies are required to examine in more detail how muscle synergies are recruited over the course of complex and continuous movements, such as learning a sequence of whole-body movements while driving a car or riding a bike. Such understanding may not only enable finding new synergy-based indices indicating the level of motor impairment in poststroke patients but also predict their recovery level along with their rehabilitation.

## Figures and Tables

**Table 1:** Patients' demographics table.

Patient no.	Sex/age	SIAS	Stroke type
P1	M/49	3	Cerebral infarction
P2	M/58	4	Cerebral infarction
P3	M/75	2	Cerebral infarction
P4	M/63	2	Brainstem infarction
P5	M/70	4	Cerebral infarction
P6	F/64	3	Acute subdural hematoma
P7	M/85	4	Cerebral infarction
P8	F/51	3	Cerebral infarction
P9	F/76	2	Cerebral infarction
P10	M/74	3	Cerebral infarction

P: patient; M: male; F: female; SIAS: stroke impairment assessment set.

## Data Availability

The recorded electromyography data from both healthy and stroke patients used to support the findings of this study are available from the corresponding author upon request.
